# Process-Oriented, Behaviorally Anchored Assessment of Clinical Reasoning in Large Language Models and the Effect of Extended Thinking: Protocol for a Prospective, Multigroup, Comparative Study

**DOI:** 10.2196/103220

**Published:** 2026-09-22

**Authors:** Adnan Agha, Muhammad Jalil, Eram Anwar, Omran Bakoush

**Affiliations:** 1 Department of Internal Medicine College of Medicine and Health Sciences United Arab Emirates University AlAin, Abu Dhabi United Arab Emirates; 2 College of Business and Economics United Arab Emirates University AlAin, Abu Dhabi United Arab Emirates

**Keywords:** clinical reasoning assessment, large language models, behaviorally anchored rating scale, LLM-as-judge, extended thinking, reasoning models, process-oriented evaluation, clinical decision support, self-preference bias, metacognition

## Abstract

**Background:**

Most clinical reasoning evaluations in large language models (LLMs) score only the final answer, usually multiple-choice accuracy, which explains little about model reasoning. Two developments stress this gap: reasoning-optimized models are now common, and several expose an explicit extended thinking control. Whether that deliberation improves the reasoning process remains untested with a validated instrument.

**Objective:**

This protocol introduces and aims to validate the Rapid Evaluation Assessment of Clinical Reasoning Tool (REACT)–AI, a Behaviorally Anchored Rating Scale (BARS) with 13 subdomains for process-oriented assessment of AI clinical reasoning, that is, the quality of the externalized reasoning rather than its faithfulness to the model’s internal computation. It also builds a conflict-of-interest–controlled LLM-as-judge pipeline and tests whether extended thinking improves reasoning quality.

**Methods:**

This 2-phase, prospective, comparative study has a within-model thinking-mode factor. Six flagship models are run in standard and extended thinking or reasoning modes, giving 12 conditions: 4 providers are toggled within the same model, and 2 pair a standard model with a dedicated reasoning model. In phase 1, 5 standardized urgent care vignettes are answered under all 12 conditions, 3 runs per condition (180 AI outputs), and by an independent expert clinician panel (n=5) and senior and junior medical students. Every output is scored by dual-blind human raters and, in parallel, by LLM judges, with no model judging its own family. Acceptance criteria for deploying the judge at scale are a weighted κ of at least 0.60 and an intraclass correlation coefficient above 0.75. In phase 2, the validated pipeline scores 3600 AI outputs. A self-correction turn and a paired Gulf English condition run alongside and are reported separately, with the AI metacognition assessment rubric and disinformation generation rate as secondary instruments.

**Results:**

As of June 2026, ethics approval was obtained (ERSC_2025_6124), and the study is registered on the Open Science Framework under embargo. Responses to the 5 phase 1 vignettes were collected from senior and junior medical students between January and June 2026; 173 scripts were received and remain sealed, unopened, and unscored. No AI outputs have been generated, and the expert panel remains unrecruited. Following the June 1, 2026, model-version lock, AI generation and scoring will begin in September 2026, with phase 1 calibration through December 2026 and phase 2 from January to April 2027; results are expected in winter 2027. The study will report the validated instrument, judge calibration against human experts, self-preference bias by model family, and the effect of extended thinking on reflection and metacognition, including null findings.

**Conclusions:**

REACT-AI and its judge pipeline are built to be reusable. They close the gap between accuracy benchmarks and genuine reasoning assessment and provide a template for studying reasoning mode effects.

**Trial Registration:**

OSF Registries osf.io/jep5t; https://osf.io/jep5t

**International Registered Report Identifier (IRRID):**

DERR1-10.2196/103220

## Introduction

### The Clinical Reasoning Gap: Process Versus Outcome

Large language models (LLMs) perform well in medical knowledge assessments. Instruction-tuned systems passed earlier state-of-the-art performance on United States Medical Licensing Examination (USMLE)–style multiple-choice questions, although human reviews still found gaps in reasoning, factuality, and safety [1]. For open-ended, board-style vignettes, accuracy can also be very high, and one comparative study reported a frontier model achieving a score of 20 out of 20 on the USMLE Step 1 cases [2]. This knowledge is clearly visible. However, a correct final answer does not prove that the reasoning is sound. The recent work shows that near-perfect benchmark accuracy can reflect pattern matching rather than reasoning and that headline benchmark scores often fail to capture real-world competence [3,4]. This distinction matters for what an instrument should measure. The Rapid Evaluation Assessment of Clinical Reasoning Tool (REACT)–AI assesses the quality of the externalized reasoning process, that is, which steps are taken and how well they are performed, rather than the faithfulness of a model’s internal computation to its stated rationale.

Clinical reasoning is a process. Clinicians gather and prioritize data, build a problem representation, weigh a differential diagnosis, plan management under uncertainty, and then reflect on their own thinking [5-8]. A scoping review of reasoning assessment screened 377 studies and concluded that the construct is multidimensional and that competence can be demonstrated only across a program of assessments targeting its separate components [6]. Management reasoning, the process of choosing among tests and treatments when the answer is not obvious, is now treated as a domain in its own right [7]. Safety concerns follow directly. A model that lands on the correct diagnosis by pattern matching can fail without warning when presented with an atypical presentation, missing data, or a case it has not encountered before. Only process-level assessment can show where these limitations lie [8].

### The Current Evaluation Landscape: Multiple-Choice Dominance and the Rise of Rubrics

Multiple-choice and short-answer accuracy still dominate the field [1], but rubric-based, process-aware evaluation is gaining ground. A randomized clinical trial assessed physicians’ diagnostic reasoning with a validated rubric that captured differential accuracy, the handling of supporting and opposing evidence, and the next diagnostic step. Access to an LLM did not improve the physicians, yet the LLM working alone beat them by 16% [9]. A randomized trial of the Articulate Medical Intelligence Explorer (AMIE) in complex cardiology used a 10-domain rubric and 3 blinded subspecialists, who preferred the AMIE-assisted assessments 46.7% of the time, compared with 32.7% for assessments by cardiologists alone [10]. Multidimensional benchmarks have followed. A proportional reasoning index applied to 21 contemporary models, several of them reasoning-optimized, found that the final-diagnosis accuracy was high but differential generation and the handling of uncertainty comparatively weak [11]. An automated reasoning evaluator assessed efficiency, factual accuracy, and completeness over 1453 structured cases and again found that critical steps were often skipped [12]. A specialist-scored headache benchmark used a 5-point rubric across 6 clinical dimensions and varied the prompting strategy [13].

This is real progress. However, each study relies on a rubric developed specifically for that study. The scales are often coarse, for example, using 3-point diagnosis scores [2], or they lack behavioral anchors, and their granularity varies considerably. None offers a validated, criterion-referenced instrument that breaks reasoning into stable behavioral subdomains that traverse across cases and models.

### The Behaviorally Anchored Rating Scale Gap

Behaviorally Anchored Rating Scales (BARSs) have a long track record in competency assessment. Rather than forming a global impression, the rater matches what is observed to explicit descriptors at each level, which trims subjectivity and lifts interrater reliability [14]. REACT brought this approach to clinical reasoning in urgent care, scoring 5 domains on a 3-point scale; across 41 simulated scenarios rated by 7 clinician-educators, it reached a Cronbach α of 0.86 and a weighted κ of 0.56 [15]. Such instruments are routinely used to assess human clinical reasoning. For AI clinical reasoning, as far as we can establish, none has been adapted. The bespoke AI rubrics described above are not anchored in the BARS framework and were not designed for comparison across studies. REACT-AI is designed to address this gap.

### Methodological Gaps in LLM-as-Judge Evaluation

Expert ratings do not scale. Scoring thousands of responses across 13 subdomains at 15-20 minutes per response would require hundreds of clinician-hours. The LLM-as-judge offers a way to address this limitation [16,17], and a recent medical-evaluation framework ran it as a jury of several automated evaluators checking outputs against expert criteria [18]. Agreement with human experts can be strong at the composite level. It tends to break down on subtle items, which may also be those with the highest clinical stakes [18,19].

Three problems hold current medical practice back. The first is monoculture. Reviews report that most studies use a single model family as the judge and apply pointwise scoring, so the field effectively relies on a single design with correlated blind spots [19]. The second is self-preference. A model can score its own family generously; on a subjective medical chat benchmark, this shifted scores by as much as 10 points, and AI judges have been shown to favor AI-written clinical plans [19-21]. The third is governance. Procedures for validating, auditing, and risk-stratifying judges remain immature, and no published medical method enforces a conflict-of-interest rule, under which no model judges its own family and judges are separated from candidates by tier [19,22]. REACT-AI answers all 3 issues with a calibrated, cross-tier judge panel and an explicit measure of self-preference.

### Extended Thinking and Reasoning Modes as a New Evaluation Dimension

A calibrated, cross-tier judge panel makes granular scoring feasible at scale, which in turn makes it possible to probe a question that the field has only recently been able to ask. A key shift since 2024 is the arrival of explicit reasoning controls. Some providers expose a per-request thinking switch that can turn deliberation on or off, set a thinking budget, or pick a reasoning-effort level. Others provide a dedicated reasoning model alongside the standard one. Across benchmarks, reasoning-optimized models tend to perform better on clinical reasoning [11,12,18]. What remains unclear is whether extended thinking improves the reasoning process rather than just the final answer. The open question is sharper still for the metacognitive processes, such as recognizing uncertainty, naming a likely bias, and marking the edge of one’s knowledge, which current evidence identifies as among the weakest aspects of AI reasoning [11]. REACT-AI scores a dedicated reflection and metacognition domain and is therefore well positioned to address this question. This provides the rationale for prespecifying hypothesis 6 and the 12-condition standard-versus-extended design.

### Objectives

This study aims to (1) develop and validate the REACT-AI instrument for process-oriented assessment of AI clinical reasoning; (2) compare clinical reasoning across 6 commercial LLMs, each in a standard and an extended-thinking or reasoning mode, against an expert clinician panel and medical students at 2 training levels; (3) validate a conflict-of-interest-controlled, cross-tier LLM-as-judge method and calibrate it against human experts; (4) quantify the Self-Preference Bias Delta across model families; (5) test the effect of Gulf English sociolinguistic variation on AI reasoning; and (6) determine whether extended thinking modes improve reasoning quality, with a prespecified focus on the reflection and metacognition domain. The 6 prespecified hypotheses that follow from these objectives, and the study phase in which each is tested, are set out in the Methods.

## Methods

### Study Design Overview

This is a prospective, multigroup comparative study. It uses a repeated-measures design with a dual-phase architecture, which we call calibrate and scale, plus a within-model thinking-mode factor (Figure 1).

**Figure 1:**
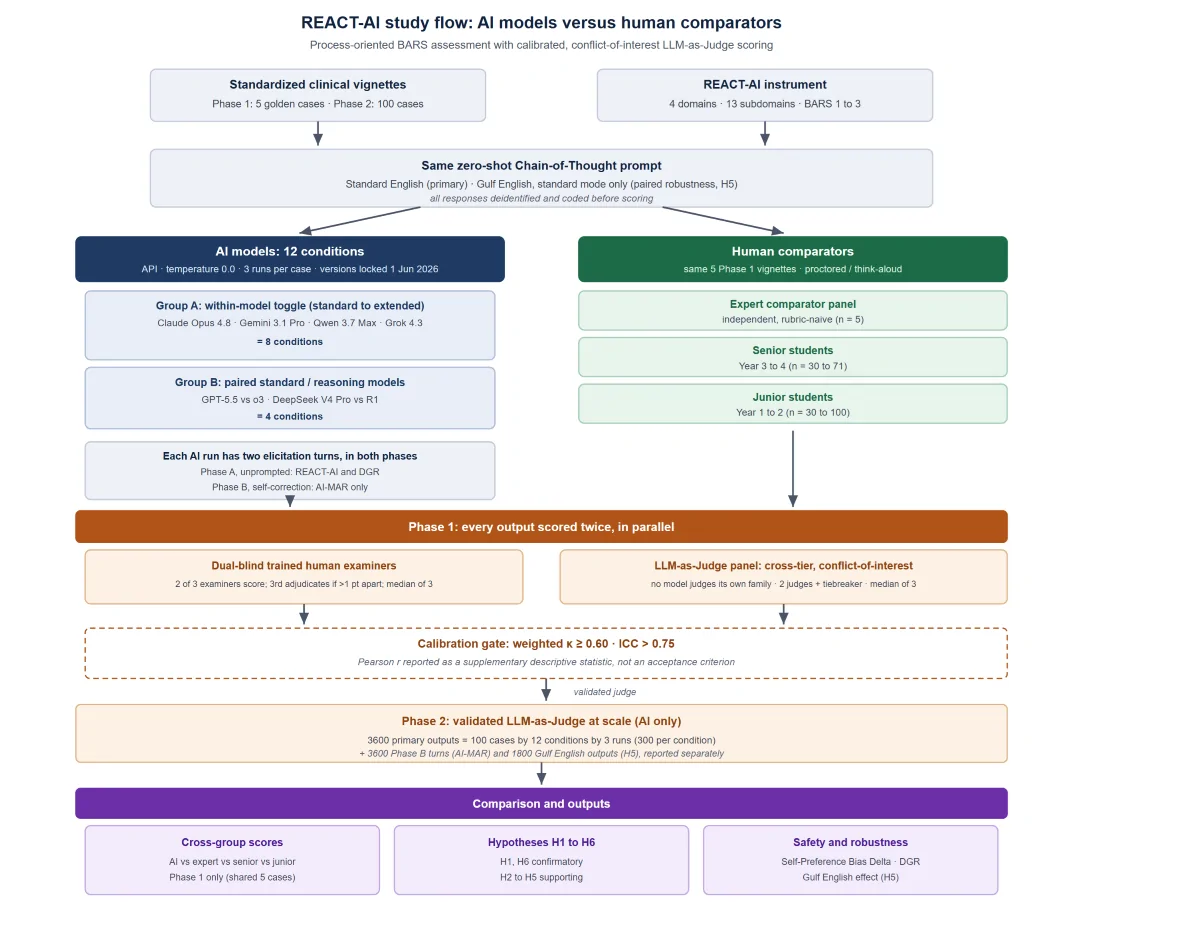
Overview of the Rapid Evaluation Assessment of Clinical Reasoning Tool (REACT)–AI study design. AI-MAR: AI Metacognition Assessment Rubric; API: application programming interface; BARS: Behaviorally Anchored Rating Scale; DGR: disinformation generation rate; ICC: intraclass correlation coefficient; LLM: large language model; REACT: Rapid Evaluation Assessment of Clinical Reasoning Tool.

#### Phase 1 (Golden Cases, Human-Scored Calibration)

Five standardized vignettes are scored by both trained human raters and LLM judges. This phase fixes the psychometric gold standard and calibrates the automated scores against human expert judgment. The prespecified acceptance criteria for deploying the automated judge at scale are a weighted κ of at least 0.60 and an intraclass correlation coefficient (ICC) above 0.75. Pearson *r* is reported as a supplementary descriptive statistic only: a correlation can remain high when a judge is systematically biased upward or downward, so agreement rather than association governs the decision to deploy.

#### Phase 2 (Proof of Efficacy, LLM-as-Judge at Scale)

A total of 100 additional vignettes are scored only by the validated judge pipeline, which provides the statistical power to assess whether the patterns hold across a wider case bank.

### Hypotheses and the Phase in Which Each Is Tested

The study tests 6 prespecified hypotheses: (1) hypothesis 1 (noninferiority): AI conditions will score noninferior to senior medical students on the overall REACT-AI, within a margin of 1 point on the 4-12 scale. (2) Hypothesis 2 (expert superiority): the independent expert comparator panel will outperform all other groups; the anticipated magnitude is approximately 2-3 points on the 4-12 scale, reported as an effect size with CIs rather than treated as a pass criterion. (3) Hypothesis 3 (domain-specific AI profile): a group-by-domain interaction will be observed in which AI conditions score relatively higher on data gathering and interpretation and relatively lower on reflection and metacognition than the expert comparator panel. The prespecified primary test is on domain profiles, that is, each group’s domain scores expressed relative to its own overall mean, thereby isolating the shape of the profile from the overall level; the same contrast on raw domain means is reported as a supporting analysis. (4) Hypothesis 4 (performance gradient): a gradient of expert over senior over junior students will be observed, with approximately 20%-30% variation among the AI systems. (5) Hypothesis 5 (Gulf English effect): Gulf English prompting will significantly reduce AI REACT-AI scores relative to standard English. (6) Hypothesis 6 (extended thinking effect): extended-thinking or reasoning modes will significantly improve scores relative to the standard mode, with the largest gain in Domain 4 (reflection and metacognition).

Human participants answer only the 5 phase 1 vignettes, whereas phase 2 adds 100 vignettes answered by the AI conditions alone. Any hypothesis that compares humans with AI is therefore estimated exclusively within phase 1, on the identical 5 cases that every group answers. Phase 2 is an AI-only benchmark and is never used for human versus AI comparison, and no contrast in this protocol is drawn across the 2 case sets. Table 1 states where each hypothesis is tested.

Hypotheses 1-3 and the human component of hypothesis 4 require the human comparator groups and are confined to the 5 phase 1 vignettes that all groups answer. Hypothesis 4 (intermodel spread), hypothesis 5, and hypothesis 6 are AI-only contrasts, estimated in both phases with phase 2 as the prespecified primary test because it carries the larger case bank.

**Table 1:** Hypotheses, comparison type, and the phase in which each is tested.

Hypothesis	Comparison	Phase tested	Case set
Hypothesis 1 noninferiority	AI versus senior students	Phase 1 only	5 shared vignettes
Hypothesis 2 expert superiority	Experts versus all other groups	Phase 1 only	5 shared vignettes
Hypothesis 3 domain profile	AI versus experts, by domain	Phase 1 only	5 shared vignettes
Hypothesis 4 performance gradient	Human gradient (expert, senior, and junior)	Phase 1 only	5 shared vignettes
Hypothesis 4 intermodel spread	Among AI conditions	Phase 2 primary, phase 1 supporting	100 vignettes (phase 2)
Hypothesis 5 Gulf English effect	Within AI conditions, paired by case	Phase 2 primary, phase 1 supporting	Paired within each phase
Hypothesis 6 extended thinking	Within AI conditions, paired by case	Phase 2 primary, phase 1 supporting	Paired within each phase

### Ethical Considerations

No patient data are used. All vignettes are standardized fictional scenarios created by clinicians from their experience of multiple clinical encounters that are not limited or identifiable with any single patient. Students and clinicians participate voluntarily and with informed consent, and institutional ethics approval is in place before any data are collected (United Arab Emirates University Social Sciences Ethics Committee approval number ERSC_2025_6124, dated April 25, 2025). The study is registered on the Open Science Framework (OSF; osf.io/jep5t), embargoed until June 2028. Registration was made under the category recording that data exist but have not been observed by the authors. Student scripts had been collected but not opened, read, or scored at the time of registration, and that remains the case at submission. Because the registration is embargoed and does not resolve publicly, an anonymized view-only link has been supplied to the editorial office so that the registered protocol can be inspected during review without lifting the embargo. The protocol is reported in line with TRIPOD-LLM (Transparent Reporting of a multivariable prediction model for Individual Prognosis or Diagnosis–LLM), the reporting guideline for studies that develop, tune, or evaluate LLMs in health care (completed checklist in Multimedia Appendix 1) [23]; any change to a model version between registration and data collection is additionally documented following CONSORT-AI (Consolidated Standards of Reporting Trials–AI) [24]. SPIRIT-AI (Standard Protocol Items, Recommendations for Interventional Trials–AI) is scoped to clinical trial protocols for a patient-facing AI intervention and does not apply to this evaluation and validation study, which has no clinical intervention or randomized allocation of patients [25].

### Experimental Conditions: 6 Models in 2 Thinking Modes

Six flagship models are each evaluated in 2 modes, a standard mode and an extended-thinking or reasoning mode, for 12 conditions in total (Table 2). Two architectures are used to create the contrast. In group A (4 providers), the contrast is a within-model toggle: the weights stay fixed and only the reasoning control changes. In group B (2 providers), the contrast pairs a standard model with the provider’s dedicated reasoning model, because those providers expose reasoning through separate end points rather than a switch. Reporting both designs is deliberate. The toggle isolates the effect of deliberation with the architecture held constant, while the paired-model comparison reflects how reasoning capability is actually shipped and used. Every condition is reached through the provider API with version-locked identifiers, a temperature set at 0.0, and 3 runs per vignette to gauge consistency. Model versions are locked as of June 1, 2026. One model is also run through its consumer web interface as a supplementary check against the API.

Qwen 3.7 Max exposes extended thinking natively, which suits the within-model toggle. Exact dated API identifiers and the Gemini thinking budget N are recorded at data collection and listed in Multimedia Appendix 2. Provider availability and version strings are reconfirmed against current provider documentation immediately before collection begins.

**Table 2:** Models, thinking modes, and API parameters (12 conditions; versions locked on June 1, 2026).

Provider	Standard-mode condition	Extended/reasoning condition	Mechanism (API parameter)
Anthropic (group A)	Claude Opus 4.8, thinking disabled	Claude Opus 4.8, thinking enabled	thinking.type = “disabled” to “enabled”
Google (group A)	Gemini 3.1 Pro, budget 0	Gemini 3.1 Pro, budget N	thinking_config.thinking_budget = 0 to N
Alibaba (group A)	Qwen 3.7 Max, thinking off	Qwen 3.7 Max, thinking on	enable_thinking = false to true
xAI^a^ (group A)	Grok 4.3, effort none	Grok 4.3, effort high	reasoning_effort = “none” to “high”
OpenAI (group B)	GPT-5.5 (standard)	o3 (reasoning model)	Separate model end points
DeepSeek (group B)	DeepSeek V4 Pro (standard)	DeepSeek R1 (reasoning model)	Separate model end points

^a^xAI: SpaceXAI.

### Participant Groups and Sample Sizes

The study compares the 12 AI conditions against 3 human comparator groups (Table 3).

Comparison groups and target sample sizes for the 2 phases. The AI arm comprises the 12 conditions (6 models in standard and extended-thinking modes, run by API at temperature 0.0, versions locked June 1, 2026), with N given as scored outputs; the human comparators are an independent expert clinician panel, distinct from the case-development, standard-setting, and scoring panels, and senior and junior medical students, with N given as participant counts. Student scripts have been collected and remain sealed, so achieved student sample sizes will be reported with the results; the expert comparator panel has not yet been recruited.

Phase 1 yields 180 primary AI outputs (5 vignettes by 12 conditions by 3 runs). Phase 2 yields 3600 primary AI outputs (100 vignettes by 12 conditions by 3 runs), which is 300 observations per condition. These headline figures count the outputs on which the primary REACT-AI score is computed, namely the Standard English, unprompted (phase A) responses. Two further streams are generated alongside them and are reported separately rather than pooled: the prompted self-correction turn (phase B), which supplies the AI Metacognition Assessment Rubric (AI-MAR) metacognition score, and the paired Gulf English condition, which supplies the hypothesis 5 robustness comparison. Table 4 sets out the full accounting.

The primary REACT-AI counts of 180 and 3600 refer to Standard English phase A outputs. Phase B adds one self-correction turn per primary output and is scored only for AI-MAR, so it adds generations without adding REACT-AI-scored outputs. The Gulf English condition is administered in standard mode only (6 conditions), paired case-by-case against the corresponding standard English output, and is analyzed under hypothesis 5 and reported separately.

Human participants generate a further set of phase 1 responses. A total of 173 student scripts were received between January and June 2026, each covering all 5 vignettes; the scripts remain sealed, so the numbers of evaluable participants and responses after exclusion of incomplete or duplicate submissions will be determined when they are opened at the start of the scoring phase. The independent expert comparator panel of 5 clinicians will contribute a further 25 responses (5 clinicians by 5 vignettes). Trained examiners score the 180 primary AI outputs and all human responses, which together form the calibration set; every response in that set is scored independently by 2 examiners. The judge panel scores all AI outputs, with 2 primary judges per output and a tiebreaker when they differ by more than one point on any subdomain.

The 4 comparator groups are chosen to span a known developmental gradient in clinical reasoning. Junior (preclinical) students, senior (clinical) students, and board-certified clinicians differ in the depth and structure of their reasoning process, not only in final-answer accuracy, so an instrument that measures reasoning quality should separate them; this contrasting-groups (known-groups) structure is itself a validity test and is the basis of hypothesis 4. Placing the AI conditions against this human gradient shows where each model sits relative to training level rather than against an absolute standard, and it follows the vignette-based, multigroup approach used in comparable protocols [26].

**Table 3:** Participant groups and sample sizes.

Group	Description	N	Selection
AI conditions (12)	6 models by 2 thinking modes, via API; versions locked on June 1, 2026; temperature 0.0	180 primary outputs (phase 1); 3600 (phase 2)	June 2026 frontier models
Expert comparator panel	Board-certified clinicians, over 10 years’ experience (internal or emergency medicine), independent of case development, standard setting, and scoring	5	Purposive; not yet recruited
Senior students	Years 3-4, clinical rotations completed	30-71 (target)	Informed consent
Junior students	Years 1-2, preclinical	30-100 (target)	Informed consent

**Table 4:** AI output accounting by stream, phase, and purpose.

Stream	Phase 1	Phase 2	Calculation	Scored for
Standard English, phase A (primary)	180	3600	Cases by 12 conditions by 3 runs	REACT^a^-AI total and domains; DGR^b^
Standard English, phase B (self-correction)	180	3600	One self-correction turn per phase A output	AI-MAR^c^ only, paired with its phase A output
Gulf English, phase A (paired robustness)	90	1800	Cases by 6 standard-mode conditions by 3 runs	REACT-AI total, for hypothesis 5 only
Total AI generations	450	9000	Sum of the 3 streams	—^d^

^a^REACT: Rapid Evaluation Assessment of Clinical Reasoning Tool.

^b^DGR: disinformation generation rate.

^c^AI-MAR: AI Metacognition Assessment Rubric.

^d^Not applicable.

### Clinical Vignettes and Prompting

Phase 1 uses 5 standardized vignettes of urgent and emergent presentations; phase 2 adds 100 more. Every participant receives the same zero-shot chain-of-thought (CoT) prompt, which asks for step-by-step reasoning across all 4 REACT-AI domains (Multimedia Appendix 2). For the Gulf English condition, a linguistically validated prompt reflects the sociolinguistic features of the United Arab Emirates health care system; it is matched to the standard English prompt on clinical content and reasoning demands so that any score difference reflects sociolinguistic variation rather than task difficulty. The Gulf English condition is administered in standard mode only, on the same vignettes as the standard English prompt in each phase, and is analyzed as a paired within-condition comparison under hypothesis 5 (Table 4). It is a prespecified robustness check rather than a third study arm, and it is not pooled into the primary REACT-AI counts. No system-level instructions, prompt engineering, or few-shot examples are added. The only deliberate change to the model behavior is the thinking-mode setting (Table 2). The prompt text remains identical across modes; any difference can be attributed to the deliberation setting rather than wording.

All vignettes are newly authored, unpublished cases written by clinicians; they are not drawn from public question banks or datasets, and the 100-case phase 2 bank is withheld until data collection is complete, which limits the risk that a model has encountered the cases during training [27]. Running each condition at temperature 0.0 with 3 independent runs quantifies output consistency, and because the AI and human groups answer the same standardized vignettes independently, there is no shared training signal or information leakage between the comparator groups. Every output is deidentified and assigned a coded identifier before scoring: human raters are blind to the source of a response (AI, senior student, junior student, or expert) and, for AI outputs, to model identity and thinking mode, and the LLM judges receive only the response text and the rubric. The self-judging shadow track that estimates the Self-Preference Bias Delta is kept separate and is the single deliberate exception to blinding. Before the calibration phase, the scoring examiners completed frame-of-reference training [28,29] and a feasibility pilot of the rating and scoring workflow, both of which have been carried out. Student scripts were collected before the scoring phase and are held sealed, and the rubric, behavioral anchors, the examiner training, and the feasibility pilot were all completed on separate practice cases and expert-authored exemplars, so no element of the instrument or its calibration was informed by any student response.

### Two-Phase AI Elicitation

Each AI run comprises 2 elicitation turns, labeled A and B to distinguish them from the 2 study phases. Both turns are run in phase 1 and phase 2.

#### Phase A: Unprompted Reasoning

The model answers with the standardized CoT prompt, and REACT-AI scoring is based on these answers alone.

#### Phase B: Prompted Self-Correction

The model is then asked to review its phase A answer and flag errors or uncertainties. AI-MAR is scored on the phase A and phase B responses as a pair, so it captures metacognition that is both spontaneous and prompted by the task. Phase B therefore adds one generation per primary output without adding a REACT-AI–scored output (Table 4).

### The REACT-AI Instrument

REACT-AI has 4 domains and 13 subdomains (Table 5), adapted from the original REACT [15]. The main changes are as follows: the Communication domain is removed, since it is not observable in text-based outputs; Domain 1 grows to 4 subdomains with the addition of understanding the presenting problem; the do-not-miss versus most-likely diagnosis distinction enters subdomain 2.3; and every behavioral anchor is rewritten for text-based assessment.

The 4 domains of the REACT-AI instrument and their 13 behaviorally anchored subdomains, adapted from REACT for text-based assessment. Each subdomain is scored from 1 (minimal) to 3 (proficient); the domain score is the average of its subdomains, and the total is the sum of the 4 domain averages, giving a range of 4.00-12.00.

**Table 5:** Rapid Evaluation Assessment of Clinical Reasoning Tool (REACT)–AI structure (4 domains and 13 subdomains).

Domain	Subdomains	Count
1. Data gathering	1.1 Understanding the presenting problem; 1.2 History elicitation; 1.3 Physical examination; 1.4 Investigations	4
2. Interpretation	2.1 Problem representation; 2.2 Differential diagnosis construction; 2.3 Reasoning/justification (do not miss vs most likely)	3
3. Management	3.1 Diagnostic strategy; 3.2 Therapeutic planning; 3.3 Contingency planning	3
4. Reflection and metacognition	4.1 Uncertainty recognition; 4.2 Cognitive bias awareness; 4.3 Self-directed learning	3
Total	—^a^	13

^a^Not applicable.

#### Instrument Development

REACT-AI was developed in 4 stages. First, the 5 REACT domains were mapped onto text-based AI assessment. Communication was removed, since it depends on spoken exchange with a patient or team and is not observable in a written model output, and the remaining 4 domains were retained. Second, each domain was expanded from a single, holistic item into behavioral subdomains, giving 13 in total, so that the instrument could discriminate between systems that cluster closely on coarser scales; Domain 1 gained the subdomain understanding the presenting problem ahead of history taking, and subdomain 2.3 was written to separate the do-not-miss from the most-likely diagnosis, a distinction with direct safety relevance. Third, every behavioral anchor was rewritten by clinician-educators to describe observable features of written reasoning rather than live simulation behavior, following the retranslation logic that underpins behaviorally anchored scales [14], and the drafted anchors were reviewed by the clinician-educator group for clinical accuracy, level discrimination, and freedom from stylistic or verbosity-related cues. Fourth, the anchors were tested in a feasibility pilot of the full rating and scoring workflow, which has been completed; the pilot was conducted on separate practice cases and expert-authored exemplars rather than on any student response, and it examined anchor clarity, scoring time, and the practicability of dual independent rating, after which the wording was refined before the calibration phase.

#### Case Development

Seven urgent care vignettes were authored and validated by the case-development panel, which also produced a consensus ideal answer for each case. Two vignettes were rejected because the panel did not reach complete consensus on the ideal answer, and the 5 cases carried forward were those for which consensus was unanimous. Requiring unanimity rather than a majority means that each retained case has an agreed reasoning pathway against which responses can be judged, and the 2 rejections are reported as part of the content-validity evidence for the case set. Because domains contain unequal numbers of subdomains, the domain score is an average rather than a sum, which prevents Domain 1 from carrying disproportionate weight in the total.

#### Separation of Clinician Roles

Sixteen clinicians are involved, in 4 panels with no overlap in membership, so that no individual authored a case, set the standard against which it was judged, answered it as a candidate, or scored responses to it in more than one capacity.

A case-development panel of 5 clinicians authored and validated the vignettes and produced the consensus ideal answers. A standard-setting and calibration panel of 3 clinicians produced the scoring rubric and the performance benchmarks and conducted examiner calibration; this panel takes no part in case authorship and does not score study responses, and its own worked answers are used as the criterion rather than scored as expert performances, since familiarity with the rubric would inflate them. An independent expert comparator panel of 5 board-certified clinicians, who took no part in case development, standard setting, or scoring and who have not seen the rubric or the ideal answers, answers the 5 vignettes under the same conditions as the students; this panel provides the expert arm for hypotheses 2 and 3 and the upper end of the hypothesis 4 gradient. A scoring panel of 3 trained examiners scores all student and AI responses and takes no part in case authorship or standard setting. The principal investigator is excluded from all 4 panels.

The design consequence is that the expert comparator is rubric-naive. A clinician who had written the anchors would know that reflection and metacognition are scored and would articulate uncertainty and bias accordingly, which would raise expert Domain 4 scores for reasons unrelated to reasoning quality and would foreclose the very question hypothesis 3 asks.

#### Examiner Training and Adjudication

The 3 scoring examiners are trained clinicians in internal medicine, emergency medicine, and related specialties. All completed frame-of-reference training [28,29], comprising conceptual orientation to behaviorally anchored scales and the 4 domains, anchor calibration with worked examples for every subdomain, and independent practice scoring of calibration cases followed by group discussion, was delivered by the standard-setting panel. A reliability gate applies: scoring of study data does not begin until the examiners reach an ICC above 0.85 on the calibration cases, with weighted kappa reported alongside. Training and calibration are complete; no study response has yet been scored.

Every response is scored independently by 2 examiners. Where the 2 differ by more than one point on any subdomain, the third examiner adjudicates, scoring the response independently against the anchors while blind to the respondent group and to the 2 primary scores, and the final subdomain score is the median of the 3. This mirrors the LLM judge panel, in which 2 primary judges score every output and a tiebreaker is invoked under the identical rule. Interrater reliability is computed on the independent scores of the 2 primary examiners, recorded before any adjudication, so that agreement is not inflated by the consensus process; the adjudicated median is used for analysis. The frequency of adjudication is reported. Reliability is monitored throughout, with the ICC recalculated every 20-50 ratings, immediate recalibration if it falls below 0.75, and independent verification of a 10% random sample.

#### Scoring

Each subdomain is scored 1 (minimal), 2 (competent), or 3 (proficient) by matching observed behavior to written behavioral anchors (Multimedia Appendix 3). The domain score is the mathematical average of the subdomain scores, not the sum. The total score is the sum of the 4 domain averages, ranging from 4.00 to 12.00. A modified percentage is then computed as ([total − 4]/8) × 100, giving a 0-100 scale. The one rule that governs everything is to match the observed behavior to the anchors rather than to a general impression.

### LLM-as-Judge Methodology

#### Overview

The judging panel operates on a conflict-of-interest-controlled rotation. No model family judges its own outputs, and the judges are drawn from a different model tier or generation from the candidate. GPT-4o and Gemini 2.5 Pro serve as judges, for instance, rather than GPT-5.5, o3, or Gemini 3.1 Pro candidates, which keeps the candidate and judge roles fully separate (Table 6). All judging uses the API with a temperature of 0.0 and structured JSON output. The judge prompt carries the full REACT-AI rubric, vignette, and model response and asks for CoT reasoning before any integer score. No reference answer is supplied, which avoids an upward pull on the scores (Multimedia Appendix 4). Two primary judges score each output. A tiebreaker is called only when the 2 reviewers differ by more than one point on any subdomain, and the final subdomain score is then the median of the 3, matching the rule used for the human examiners. Interjudge reliability is computed on the 2 primary judges’ scores before any tiebreaker is applied. Scoring is issued as 2 separate judge calls so that the primary outcome cannot be contaminated by the secondary instrument. The first call is shown the Phase A response alone and returns the 13 REACT-AI subdomain scores and the Disinformation Generation Rate (DGR) flags; the second call is shown the Phase A and Phase B responses together and returns the AI-MAR dimensions. Without this separation, a judge scoring Phase A reasoning would also be reading the model’s own subsequent critique of it, which could raise or lower the REACT-AI score for reasons unrelated to the reasoning being scored.

The judge models assigned to each candidate under the conflict-of-interest rotation. No model judges its own family, and judges sit one tier or generation below the candidates. Two primary judges score every output, with the tiebreaker invoked only when they differ by more than one point on any subdomain.

**Table 6:** Judge rotation matrix (conflict-of-interest and cross-tier).

Candidate scored (provider)	Judge 1	Judge 2	Tiebreaker
GPT-5.5/o3 (OpenAI)	Gemini 2.5 Pro	Claude Sonnet 4.6	DeepSeek V3
Gemini 3.1 Pro (Google)	GPT-4o	Claude Sonnet 4.6	DeepSeek V3
Claude Opus 4.8 (Anthropic)	GPT-4o	Gemini 2.5 Pro	DeepSeek V3
DeepSeek V4 Pro / R1 (DeepSeek)	Gemini 2.5 Pro	Claude Sonnet 4.6	GPT-4o
Grok 4.3 (SpaceXAI)	Gemini 2.5 Pro	GPT-4o	Claude Sonnet 4.6
Qwen 3.7 Max (Alibaba)	Gemini 2.5 Pro	Claude Sonnet 4.6	GPT-4o

#### Self-Preference Bias Delta

A parallel shadow track records the output of each model. The delta is the difference between a model’s mean score when judging itself and the mean score from external judges on the same outputs, reported with 95% CIs. Given prior evidence of self-enhancement and evaluator-identity effects [19-21], we treat this as a finding worth reporting in its own right rather than a confound to be removed. To guard against inadvertently validating an instrument that favors one style of reasoning, the behavioral anchors describe clinical reasoning behaviors rather than linguistic form or verbosity, judges are instructed to score the observed evidence against the anchors and not to reward surface fluency, and the cross-tier rotation together with the self-preference bias delta is designed to surface any stylistic favoritism.

### Secondary Instruments

#### AI-MAR

Five dimensions are scored 1-3 across both elicitation phases, namely spontaneous uncertainty expression, error detection, confidence calibration, knowledge-boundary recognition, and reasoning-process awareness (Multimedia Appendix 5).

#### DGR

The share of clinically incorrect or fabricated statements per response, scored by the calibrated judge panel and audited against human raters in phase 1.

### Statistical Analysis

#### Primary Analysis (Phase 1)

A linear mixed effects model of the modified REACT-AI score, with fixed effects for Group, Case, and their interaction, and random intercepts for the response nested within participant and for rater:

Score ~ Group + Case + Group by Case + (1 | ParticipantID / ResponseID) + (1 | RaterID)

The case is modeled as a fixed effect because the 5 phase 1 vignettes are purposively selected and exhaustive rather than a sample from a larger pool, so a case random intercept is not additionally identifiable alongside the fixed case term. ResponseID uniquely identifies a single answer, which is one participant’s answer to one case or one AI condition’s run on one case; nesting it within participant represents the 3 repeated runs per AI condition and the repeated cases per student, and it also accounts for the 2 independent ratings of every response, so that ratings are not treated as independent observations.

#### Analysis at Scale (Phase 2)

In phase 2, the 100 vignettes are a sample from a larger case bank, so the case is modeled as a random effect:

Score ~ Model by Mode by Domain + (1 | CaseID) + (1 | CaseID: Condition) + (1 | ResponseID) + (1 | JudgeID)

The CaseID by Condition term represents responses nested within condition and case, ResponseID represents the repeated runs and the 4 domain scores drawn from a single response, and JudgeID mirrors the rater term in phase 1.

#### Thinking-Mode Analysis (Hypothesis 6)

Hypothesis 6 is estimated on the AI conditions using the phase 2 model above, in which mode (standard versus extended) is a within-condition factor, with the equivalent phase 1 fit as a supporting analysis. The prespecified test of hypothesis 6 is the main effect of mode and the mode-by-domain interaction, with a planned contrast that isolates the mode effect on Domain 4 (reflection and metacognition) and tests that the Domain 4 gain exceeds the mean gain across Domains 1-3. Group A (toggle) and Group B (paired-model) conditions are analyzed both together and as prespecified subgroups, since the 2 designs support different causal readings.

#### Domain-Level, Gulf English, and Calibration Analyses

A separate mixed effects model is fitted for each domain. The Gulf English effect is tested within the AI conditions by paired comparison (Wilcoxon signed-rank or paired *t* test). Judge calibration uses ICC(2,1) and weighted Cohen kappa on the 180 primary phase 1 outputs, with acceptance set at weighted κ of at least 0.60 and ICC above 0.75. Pearson *r* is reported as a supplementary descriptive statistic and is not used as an agreement criterion, because a correlation can remain high in the presence of systematic upward or downward judge bias, which is precisely what a calibration check must detect. Interjudge reliability and how often the tiebreaker is needed are also reported. If a subdomain fails to meet the acceptance criteria (weighted κ below 0.60 or ICC at or below 0.75), that subdomain reverts to human-only scoring in phase 2; if failures are widespread, additional golden cases are added and the judge is recalibrated before phase 2 proceeds.

#### Multiplicity

A single, tiered scheme controls the error rate; no 2 corrections are applied to the same comparison.

##### Tier 1: Confirmatory

Two end points carry the study’s primary claims: hypothesis 1, the noninferiority of the AI conditions compared with senior medical students, and hypothesis 6, the main effect of mode together with the prespecified Domain 4 contrast. Each is tested at α=.05, with the Holm step-down procedure controlling the family-wise error rate across the 2. Hypothesis 1 is a one-sided noninferiority test against a prespecified margin and is reported with its CI relative to that margin; it is not part of any pairwise superiority family. The noninferiority margin is 1 REACT-AI point or less on the 4-12 scale, equivalently 12.5 or less on the 0-100 scale, based on the approximately 1-point scoring variability in the original REACT validation [15].

##### Tier 2: Supporting

Hypotheses 2-5, together with all pairwise between-group contrasts, are tested within families using the Tukey honestly significant difference (HSD) test, which controls the family-wise error rate across all pairwise comparisons of group means by using the studentized range distribution. With the AI conditions represented in standard mode, the between-group family covers 9 groups (6 AI models plus expert, senior, and junior students) and therefore 36 pairwise comparisons. Comparisons on the total score form one family, and each domain-level analysis forms its own family. No further Bonferroni adjustment is applied on top of the Tukey HSD: Tukey already controls the family-wise error rate at 0.05 for this comparison set, and adding a second correction would reduce the realized error rate far below the nominal level and inflate the type II error rate correspondingly.

##### Tier 3: Exploratory

All remaining analyses, including the factor structure, the association between AI-MAR and Domain 4, the relationship between DGR and overall performance, case-difficulty effects, the API versus consumer interface sensitivity check, temporal stability across the phase 2 case bank, and a comparison of the standard-setting panel’s worked answers with the independent expert comparator panel’s responses as a descriptive estimate of the effect of rubric familiarity on expert scores, are reported without adjustment and are labeled exploratory. They are distinguished throughout from the confirmatory and supporting tests and are not used to support any prespecified claim.

#### Missing and Incomplete Data

The mixed effects models use all available data and handle unbalanced designs without imputation. Students who complete fewer than 3 of the 5 vignettes are excluded from the primary analysis but retained in a sensitivity analysis; those who withdraw consent are excluded entirely. For AI outputs, API errors, timeouts, or refusals are retried up to 3 times and, if unresolved, scored 1 (minimal) across subdomains and tagged so that a safety-motivated refusal can be separated from a reasoning failure in a sensitivity analysis. A rater whose calibration ICC falls below 0.60 is retrained and, if reliability remains low, replaced.

#### Sample Size and Validity

The student sample, for which a maximum of 171 participants was targeted, provides over 95% power to detect a medium effect (Cohen *d* of 0.5) and over 85% power for a small-to-medium effect (*d=*0.35) at α=.05, the repeated-measures design (5 cases per participant) adds an estimated 50%-70% power over independent measures, and the sample exceeds the minimum of 22 needed for stable ICC estimates. Formal factor analysis is not undertaken in phase 1, where it would be underpowered; construct validity rests on the contrasting-groups gradient (hypothesis 4) and on content validity from the REACT lineage and expert-anchored rewriting, with internal consistency assessed by Cronbach alpha. A total of 173 student scripts were received, slightly above the target maximum, which is consistent with a small number of duplicate or incomplete submissions; these are identified and removed when the sealed scripts are opened, and the power statements above refer to the evaluable sample, which will be reported with the results.

#### Exploratory Factor Structure and Reliability (Phase 2)

The phase 2 data provide 3600 primary observations across the 13 subdomains, which is sufficient for an exploratory examination of the instrument structure. We will report sampling adequacy (Kaiser-Meyer-Olkin) and Bartlett test of sphericity, the extraction method and the criteria used to retain factors (parallel analysis, the scree plot, and eigenvalues), the rotation applied (oblique, since the 4 domains are expected to correlate), and the full pattern matrix of subdomain loadings with the variance explained. Reliability will be reported alongside the structure rather than separately, as Cronbach alpha and McDonald omega for the total scale and for each domain, with the ICC for interrater and interjudge agreement. Because the observations are clustered within cases and conditions, and because judge-derived scores are not independent of the calibration step, this analysis is exploratory and is reported as evidence about the instrument rather than as a confirmatory test of a hypothesized factor structure. Phase 2 provides 300 observations per condition (100 cases by 3 runs), which supports precise estimates of the differences between the models and modes.

### Registration and Amendments

#### Amendment 1: Summary

Relative to the original OSF registration, this protocol (1) expands the design from 5 single-mode models to 12 conditions, namely 6 models each evaluated in a standard and an extended-thinking or reasoning mode; (2) introduces the Group A toggle (Claude Opus 4.8, Gemini 3.1 Pro, Qwen 3.7 Max, Grok 4.3) versus Group B paired-model (GPT-5.5 and o3 and DeepSeek V4 Pro and R1) distinction; (3) adds hypothesis 6 on the effect of extended thinking, with a prespecified planned contrast on the reflection and metacognition domain; (4) updates output volumes to 180 in phase 1 and 3600 in phase 2; (5) extends the conflict-of-interest judge rotation to all 6 providers, with judges separated from candidates by tier; and (6) adds the within-subjects Mode factor to the analysis plan. The primary instrument, calibration targets, secondary instruments (AI-MAR and DGR), self-preference bias delta, the Gulf English manipulation, and human comparison groups remain unchanged. The model versions are locked on June 1, 2026.

#### Amendment 2: Summary

Following peer review, and before any data collection, a second amendment records the following changes to the registered analysis plan: (1) the phase 1 primary model drops the case random intercept, which was not identifiable alongside the fixed case term, and adds response-level nesting so that repeated runs and the 2 independent ratings per response are represented; (2) the phase 2 model adds response-level and judge random effects; (3) a single tiered multiplicity scheme replaces the combination of Tukey HSD and Bonferroni correction, with hypothesis 1 and hypothesis 6 as confirmatory end points under Holm, supporting comparisons under Tukey within families, and exploratory analyses labeled as such; (4) Pearson *r* is recorded as a supplementary descriptive statistic rather than a calibration target, leaving weighted kappa and ICC as the acceptance criteria; (5) the Gulf English condition is specified as standard mode only, administered in both phases and analyzed as a paired within-condition comparison; (6) judging is issued as 2 separate calls, so that REACT-AI and DGR are scored on the Phase A response alone and AI-MAR on the Phase A and Phase B pair; (7) the instrument development history, the 4-panel separation of clinician roles, and the examiner adjudication rule are stated explicitly; (8) the expert comparator panel is specified as 5 independent, rubric-naive clinicians, distinct from the case-development and standard-setting panels; (9) Hypothesis 2 is restated so that superiority is the test and the 2-3 point magnitude is an anticipated effect size rather than a pass criterion, and hypothesis 3 is restated explicitly as a group-by-domain interaction tested on domain profiles; and (10) the status of the student scripts collected between January and June 2026 is recorded. No hypothesis, instrument, comparator group, output volume, or model version has been added, removed, or altered. The amendment is filed as an OSF registration update before the start of data collection in September 2026.

## Results

As of submission (June 2026), no response of any kind has been opened, read, deidentified, scored, or analyzed, and no AI outputs have been generated.

Raw free-text responses to the 5 phase 1 vignettes were collected from senior and junior medical students between January and June 2026 under the approved protocol, a period that spanned the May 30, 2026, registration date. A total of 173 scripts were received. The scripts are held sealed and will be opened, deidentified, and coded only when the scoring phase opens in September 2026, so the number of evaluable participants after exclusion of incomplete or duplicate submissions is not yet known and will be reported with the results. The OSF registration discloses this status, recording that student responses existed but had not been assessed, scored with REACT-AI, or analyzed; that remains the case. The independent expert comparator panel has not yet been recruited, and no expert comparator responses have been collected. Case development, standard setting, and examiner calibration were complete.

Institutional ethics approval is in place (United Arab Emirates University Social Sciences Ethics Committee, ERSC_2025_6124, dated April 25, 2025), and the study is registered on the OSF and embargoed until June 2028. The article processing charge is covered by the College of Medicine and Health Sciences, United Arab Emirates University. Following the June 1, 2026, model-version lock, AI generation and all scoring will begin in September 2026, with phase 1 calibration from September to December 2026 and the phase 2 benchmark from January to April 2027; analysis is planned for the first half of 2027, and results are expected in winter 2027. All prespecified outcomes below, including any null results, will be reported in full.

Phase 1 will yield (1) the psychometric properties of REACT-AI (Cronbach alpha, ICC, weighted kappa), (2) comparative REACT-AI scores across all groups, with domain-level profiles, (3) calibration metrics for how closely LLM judges track human raters, including where agreement falls off on the more nuanced subdomains, (4) effect sizes for the Gulf English condition. Phase 2 will yield (5) 100-case benchmark scores for all 12 conditions across the 13 subdomains, (6) self-preference bias delta values for each model family, (7) DGR safety metrics, (8) AI-MAR metacognition profiles from the paired Phase A and Phase B responses, (9) standard-versus-extended thinking contrasts, including the planned Domain 4 contrast, and (10) the exploratory factor structure of the instrument with its accompanying reliability estimates.

The prespecified hypotheses (hypothesis 1-6) and the phase in which each is tested, are stated in the Methods (Table 1); each will be tested and reported, including any null result. Comparisons between the human groups and the AI conditions are confined to phase 1, where all groups answer the same 5 vignettes, and the phase 2 benchmark is reported as an AI-only result. Current evidence suggests that AI reasoning is strongest on data handling and final-diagnosis accuracy and weakest in differential breadth and the navigation of uncertainty [9,11,12], so comparatively high scores are anticipated in Domains 1 and 2 and lower scores in Domain 4. If deliberation genuinely improves metacognition, and not only the final answer, extended thinking should lift Domain 4 scores the most.

## Discussion

### Principal Findings

This is a protocol; therefore, the findings are anticipated rather than observed, and every prespecified outcome, including any null result, will be reported in full. If the instrument performs as designed, REACT-AI should separate the comparator groups along the novice-to-expert gradient (hypothesis 4), with board-certified clinicians scoring highest and an expert advantage of approximately 2-3 points on the 4-12 scale (hypothesis 2), and with the AI conditions scoring at least as well as senior students overall (hypothesis 1). We anticipate a characteristic AI profile that is comparatively strong on data gathering and interpretation but weaker on reflection and metacognition (hypothesis 3), mirroring evidence that current models reason well toward a final answer but handle uncertainty and self-monitoring less well [11]. We expect Gulf English prompting to lower AI scores relative to standard English (hypothesis 5), consistent with reports that sociodemographic and linguistic framing shifts model behavior [30]. Finally, we expect extended thinking to raise scores, with the largest gain in the reflection and metacognition domain (hypothesis 6); a null or reversed effect here would itself be informative, since it would show that extra deliberation improves the answer without improving the reasoning process. Calibration is expected to reach the acceptance criteria on most subdomains, with any subdomain that falls short scored by humans only.

### Comparison With Prior Work

Process-aware evaluation is not new, but the previous work has been narrow. The diagnostic-reasoning trial validated its rubric, then applied it to a single model, and never used a behaviorally anchored scale [9]. The AMIE cardiology trial relied on 10 domains and 3 human subspecialists, an approach that does not scale [10]. Large multimodel benchmarks scored many systems, yet each used its own unanchored index [11,12]. Automated medical evaluation has reached for LLM juries, but without conflict-of-interest controls or anchored scoring [18]. Recent work has also cautioned that headline benchmark accuracy can overstate real competence, because models may pattern-match rather than reason and because current evaluations often fail to capture real-world performance [3,4]. No prior study has combined a validated, behaviorally anchored instrument, cross-tier judging with an explicit conflict-of-interest rule, a standard-versus-extended contrast, and a sociolinguistic stress test. REACT-AI is built to fill that gap, and its 13 subdomains make specific strengths and weaknesses visible in a way that supports targeted model improvement and sensible deployment decisions.

### Strengths and Limitations

The main strengths are the behaviorally anchored instrument, which trims rater subjectivity [14]; the calibrate-and-scale design, which buys psychometric rigor on a small gold-standard set before extending throughput across a large case bank [18]; the conflict-of-interest, cross-tier judge rotation with a reported self-preference bias delta, which addresses the monoculture and self-preference problems that are common and largely unaddressed in medical LLM-as-judge work [17,19-22]; and a preregistered, hypothesis-driven analysis plan. Several limitations temper these. REACT-AI is adapted from a tool validated for urgent care, so its fit for chronic-care reasoning may be limited. Frontier models move quickly, and version-locking captures a snapshot. The toggle design (Group A) and the paired-model design (Group B) are not perfectly comparable, which is why they are analyzed both together and as prespecified subgroups. Deployment of the automated judge depends on reaching adequate human agreement in phase 1; any subdomain that misses the calibration criteria is scored by humans only. Gulf English is one dimension of sociolinguistic variation among many, it is tested in standard mode only, so any interaction between prompt language and thinking mode is outside the registered design, and the study evaluates text-based outputs, so it assesses the quality of the externalized reasoning rather than the faithfulness of a model’s internal computation to its stated rationale. The exploratory factor analysis (EFA) is conducted on clustered, judge-derived phase 2 scores and is therefore evidence about the instrument rather than a confirmatory structural validation. The expert comparator panel comprises 5 clinicians, which anchors the novice-to-expert gradient but makes the expert contrasts the least precise in the study; the anticipated 2-3 point expert advantage is therefore reported with CIs rather than as a threshold, and the domain-profile interaction that tests hypothesis 3 rests on 5 expert profiles and is interpreted with corresponding caution. Student scripts were collected before the scoring phase and were held sealed, which prevents outcome knowledge from influencing instrument development but also means that the achieved sample and its completeness were not known when this protocol was written.

### Future Directions

If the instrument calibrates well, the validated judge pipeline can be reused to track reasoning quality across model releases without repeating the full human-scoring step, and the anchors can be extended to chronic-care and specialty presentations. The self-preference bias delta framework can be applied to other evaluator panels, and the standard-versus-extended contrast can be repeated as reasoning controls mature. The same instrument, judge rotation, and scoring formulas are intended to carry into the results paper so that the protocol and its findings can be compared directly.

### Dissemination Plan

The deidentified data, the full REACT-AI rubric and behavioral anchors, CoT and judge prompts, and the analysis code will be released on the OSF upon publication, and the results will be reported in a peer-reviewed paper that reports every prespecified hypothesis, including null findings. A living open-access REACT-BARS leaderboard is planned so that new models can be scored against the same instrument over time. Findings will also be shared with the biomedical informatics and medical education communities through conference presentation.

### Conclusions

This protocol sets out a process-oriented approach to assessing AI clinical reasoning, one that prioritizes the quality of the reasoning over the accuracy of the final answer and that is designed to measure, for the first time with a validated process instrument, whether extended thinking improves the reasoning process itself. REACT-AI, its conflict-of-interest–controlled judge pipeline, and the self-preference bias delta framework are intended as reusable, open-access resources for the biomedical informatics community. Whether the prespecified expectations hold is an empirical question that the study is designed to answer, and all outcomes, including null results, will be reported.

## Appendix

Multimedia Appendix 1 TRIPOD-LLM reporting checklist.

Multimedia Appendix 2 Standardised Chain-of-Thought prompts.

Multimedia Appendix 3 REACT-AI scoring rubric (behavioural anchors).

Multimedia Appendix 4 LLM-as-Judge prompt templates and JSON schemas.

Multimedia Appendix 5 AI Metacognition Assessment Rubric (AI-MAR).

## Abbreviations

AI-MAR: AI Metacognition Assessment Rubric
AMIE: Articulate Medical Intelligence Explorer
BARS: Behaviorally Anchored Rating Scale
CONSORT-AI: Consolidated Standards of Reporting Trials–AI
CoT: chain-of-thought
DGR: disinformation generation rate
EFA: exploratory factor analysis
HSD: honestly significant difference
ICC: intraclass correlation coefficient
LLM: large language model
OSF: Open Science Framework
REACT: Rapid Evaluation Assessment of Clinical Reasoning Tool
SPIRIT-AI: Standard Protocol Items, Recommendations for Interventional Trials–AI
TRIPOD-LLM: Transparent Reporting of a multivariable prediction model for Individual Prognosis or Diagnosis–Large Language Model
USMLE: United States Medical Licensing Examination

## Data Availability

The datasets generated and analyzed during this study will be available in the Open Science Framework repository upon publication, together with the full scoring rubric, prompts, judge templates, and analysis code.
