# Supplementary material for: Process-Oriented, Behaviorally Anchored Assessment of Clinical Reasoning in Large Language Models and the Effect of Extended Thinking: Protocol for a Prospective, Multigroup, Comparative Study
Source: JMIR Res Protoc. 2026 Sep 22;15:e103220. doi: 10.2196/103220 (PMC13643257; doi:10.2196/103220)
Supplement: Supplementary file 1 [file resprot_v15i1e103220_app1.pdf]

Multimedia Appendix 1. TRIPOD-LLM reporting checklist

This protocol is reported in line with the TRIPOD-LLM statement, the reporting guideline for studies that develop, tune, prompt-engineer, or evaluate large language models in healthcare [1]. The full checklist comprises 19 main items and 50 subitems across the title, abstract, introduction, methods, open science practices, patient and public involvement, results, and discussion; under the modular format of the guideline, the applicable items depend on the research design and task.

Research design (this study): LLM evaluation and instrument development. **LLM task:** process-oriented assessment (scoring) of clinical reasoning. The item descriptions below are paraphrased; the authoritative wording and a fillable version are available from the official TRIPOD-LLM tool. Because this is a study protocol, items that concern observed results are marked as pre-specified in the analysis plan and to be completed in the results paper.

Section	Item	Reporting topic (paraphrased)	Location in manuscript
Title	1	Identify the work as an LLM evaluation and instrument-development study, naming the task, the population, and the target construct.	Title page
Abstract	2	Structured summary of background, objectives, methods, status, and planned outcomes.	Abstract
Introduction	3	Background and rationale, including the clinical need and the gap addressed.	Introduction (The clinical reasoning gap; Methodological gaps in LLM-as-Judge evaluation)
Introduction	4	Specific objectives and pre-specified hypotheses.	Introduction (Objectives); Methods (Hypotheses and the phase in which each is tested; Table 1)
Methods	5	Source and eligibility	Methods (Clinical

Section	Item	Reporting topic (paraphrased)	Location in manuscript
		of the cases, how they were obtained, and steps taken to avoid data contamination or leakage.	vignettes and prompting)
Methods	6	LLMs evaluated, including provider, model identifiers, version lock, access route, and settings.	Methods (Experimental conditions; Table 2)
Methods	7	Prompting strategy, including the standardised zero-shot chain-of-thought prompt, the Gulf English variant, and the self-correction turn.	Methods (Clinical vignettes and prompting; Two-phase AI elicitation); Multimedia Appendix 2
Methods	8	Outcome definition and scoring instrument, including domains, sub-domains, behavioural anchors, and scoring formulas, with the instrument development history.	Methods (The REACT-AI instrument; Instrument development; Table 5); Multimedia Appendix 1
Methods	9	Evaluation and analysis methods, including the judge panel, conflict-of-interest rotation, calibration metrics and acceptance criteria, statistical models, and the multiplicity scheme.	Methods (LLM-as-Judge methodology; Statistical analysis; Table 6); Multimedia Appendix 3
Methods	10	Human oversight, panel roles, examiner	Methods (Separation of clinician roles;

Section	Item	Reporting topic (paraphrased)	Location in manuscript
		training, adjudication, and blinding.	Examiner training and adjudication; Clinical vignettes and prompting)
Methods	11	Sample size and its justification, including power and reliability considerations, and the output accounting.	Methods (Sample size and validity; Table 4)
Methods	12	Handling of missing, incomplete, or failed responses, and of unreliable raters.	Methods (Missing and incomplete data)
Open science	13	Registration, protocol availability, and plans to share data, code, prompts, and the instrument.	Methods (Ethics and registration; Registration and Amendment 1, Amendment 2); Declarations (Data availability); Discussion (Dissemination plan)
Patient and public involvement	14	Whether and how patients or the public were involved.	Not applicable: no patient data are used and no patients or members of the public were involved; all vignettes are standardised fictional cases (Methods, Ethics and registration)
Results	15	Participants and flow, including numbers assessed at each stage.	Results (Current status and timeline); Figure 1. Student scripts (n = 173) were collected between January and June 2026 and are held

Section	Item	Reporting topic (paraphrased)	Location in manuscript
			sealed and unopened; evaluable numbers and full participant flow will be reported in the results paper.
Results	16	Main performance and comparison results, with uncertainty.	Results (planned outputs a to j). Pre-specified; to be reported in the results paper.
Results	17	Additional analyses, including subgroup, sensitivity, and exploratory analyses.	Methods (Statistical analysis, Tier 3 exploratory; Exploratory factor structure and reliability). To be reported in the results paper.
Discussion	18	Limitations, including generalisability and the process-versus-faithfulness boundary.	Discussion (Strengths and limitations)
Discussion	19	Interpretation, implications, and comparison with prior work.	Discussion (Principal findings; Comparison with prior work; Conclusions)

Note. As a study protocol, this checklist documents where each reporting item is addressed in the protocol and the pre-specified analysis plan. Items concerning observed results (15 to 17) are pre-specified here and will be completed in the peer-reviewed results paper, in line with the registered plan on the Open Science Framework (osf.io/jep5t). Reporting also draws on CONSORT-AI for the documentation of any model-version change between registration and data collection [2]. SPIRIT-AI is scoped to clinical trial protocols for a patient-facing AI intervention and does not apply to this evaluation and validation study, which has no clinical intervention and no randomised allocation of patients [3].

References

1. Gallifant J, Afshar M, Ameen S, et al. The TRIPOD-LLM reporting guideline for studies using large language models. Nat Med. 2025;31(1):60-69. doi:10.1038/s41591-024-03425-5.

2. Liu X, Cruz Rivera S, Moher D, Calvert MJ, Denniston AK. Reporting guidelines for clinical trial reports for interventions involving artificial intelligence: the CONSORT-AI extension. Nat Med. 2020;26(9):1364-1374. doi:10.1038/s41591-020-1034-x.

3. Cruz Rivera S, Liu X, Chan AW, Denniston AK, Calvert MJ. Guidelines for clinical trial protocols for interventions involving artificial intelligence: the SPIRIT-AI extension. Nat Med. 2020;26(9):1351-1363. doi:10.1038/s41591-020-1037-7.
