# Supplementary material for: Process-Oriented, Behaviorally Anchored Assessment of Clinical Reasoning in Large Language Models and the Effect of Extended Thinking: Protocol for a Prospective, Multigroup, Comparative Study
Source: JMIR Res Protoc. 2026 Sep 22;15:e103220. doi: 10.2196/103220 (PMC13643257; doi:10.2196/103220)
Supplement: Supplementary file 2 [file resprot_v15i1e103220_app2.pdf]

Multimedia Appendix 2. Standardised Chain-of-Thought prompts

Version-locked API identifiers (recorded at data collection; locked on 1 June 2026). Anthropic claude-opus-4-8 (thinking disabled or enabled); Google gemini-3.1-pro (thinking_budget 0 or N); Alibaba qwen-3.7-max (enable_thinking false or true); xAI grok-4.3 (reasoning_effort none or high); OpenAI gpt-5.5 and o3; DeepSeek deepseek-v4-pro and deepseek-r1. All at temperature 0.0. Exact dated strings are fixed immediately before collection and reported with the results.

Standard English Chain-of-Thought prompt (Phase A, unprompted reasoning). "You are presented with a clinical scenario. Think step by step and show your reasoning across four areas: (1) gathering and prioritising data; (2) interpreting the data to form a problem representation and a ranked differential diagnosis, separating the most likely diagnosis from do-not-miss possibilities; (3) management, including a diagnostic strategy, initial treatment, and contingency planning; and (4) reflection, including your uncertainty, any cognitive biases at play, and what further information you would seek. Give your full reasoning."

Gulf English Chain-of-Thought prompt. A linguistically validated rendering of the identical task that reflects United Arab Emirates healthcare sociolinguistic features, holding the clinical content and the four-area structure constant. The two prompts are matched on clinical content and reasoning demands; therefore, any score difference reflects sociolinguistic variation rather than task difficulty. The Gulf English prompt is administered in standard mode only, on the same vignettes as the Standard English prompt in each phase, and is analysed as a paired within-condition comparison under H5.

Self-correction prompt (Phase B, prompted self-correction). "Review your previous response. Identify any errors, omissions, or points of uncertainty, and revise your reasoning if appropriate."

Administration. Every condition receives the identical prompt text; the only deliberate change to model behaviour is the thinking-mode setting. No system-level instructions, prompt engineering, or few-shot examples are added, which preserves the zero-shot design. Each run uses a fresh API session with no prior context. Phase A and Phase B are both run in Phase 1 and in Phase 2. REACT-AI and the Disinformation Generation Rate are scored on the Phase A response alone; the AI Metacognition Assessment Rubric is scored on the Phase A and Phase B responses as a pair.

References

1. Peterson BD, Magee CD, Martindale JR, et al. REACT: Rapid Evaluation Assessment of Clinical Reasoning Tool. J Gen Intern Med. 2022;37(9):2224-2229. doi:10.1007/s11606-022-07513-5.
