# Supplementary material for: Process-Oriented, Behaviorally Anchored Assessment of Clinical Reasoning in Large Language Models and the Effect of Extended Thinking: Protocol for a Prospective, Multigroup, Comparative Study
Source: JMIR Res Protoc. 2026 Sep 22;15:e103220. doi: 10.2196/103220 (PMC13643257; doi:10.2196/103220)
Supplement: Supplementary file 3 [file resprot_v15i1e103220_app3.pdf]

Multimedia Appendix 3. REACT-AI scoring rubric (behavioural anchors)

Each sub-domain is scored 1 (Minimal), 2 (Competent) or 3 (Proficient). The domain score is the average of its sub-domain scores. The total is the sum of the four domain averages (4.00 to 12.00). The modified score is ((Total minus 4) divided by 8) times 100, giving a 0 to 100 scale. Domain scores are averaged, never summed, because the domains contain unequal numbers of sub-domains.

The instrument is adapted from the validated REACT tool [1], and the anchors follow the retranslation logic used in the construction of behaviourally anchored rating scales [2].

Domain 1: Data Gathering

1.1 Understanding the Presenting Problem

1, Minimal: Restates the chief complaint with little framing and misses the clinical question or its urgency.

2, Competent: Frames the presenting problem and identifies the central clinical question and its time sensitivity.

3, Proficient: Frames the problem in illness script terms, states the key question and urgency, and notes what would change management.

1.2 History Elicitation

1, Minimal: Requests few or generic history elements and omits pertinent positives and negatives.

2, Competent: Requests a structured, relevant history covering the most pertinent positives and negatives.

3, Proficient: Pursues a hypothesis-driven history, including discriminating features that separate competing diagnoses.

1.3 Physical Examination

1, Minimal: Names no examination steps or only non-specific ones.

2, Competent: Specifies relevant and focused examination components tied to the presentation.

3, Proficient: Prioritises high-yield, discriminating findings and states how each would be interpreted.

1.4 Investigations

1, Minimal: Orders few or untargeted tests or an indiscriminate panel.

2, Competent: Selects appropriate first-line investigations matched to the leading hypotheses.

3, Proficient: Sequences investigations by pre-test probability and by their expected effect on management, avoiding low-yield testing.

Domain 2: Interpretation

2.1 Problem Representation

1, Minimal: Offers no coherent summary; data are presented as a list.

2, Competent: Produces a concise problem representation that integrates the key features.

3, Proficient: Produces an abstracted, semantically qualified problem representation that narrows the differential.

2.2 Differential Diagnosis Construction

1, Minimal: Offers a single diagnosis or an unranked, narrow list.

2, Competent: Offers a ranked differential covering the most likely conditions.

3, Proficient: Offers a prioritised, suitably broad differential with explicit discriminating features.

2.3 Reasoning and Justification (Do Not Miss versus Most Likely)

1, Minimal: Does not justify the diagnosis or separate dangerous from likely conditions.

2, Competent: Justifies the leading diagnosis and names at least one do-not-miss alternative.

3, Proficient: Separates the most likely diagnosis from do-not-miss possibilities, justifying each with evidence and stated trade-offs.

Domain 3: Management

3.1 Diagnostic Strategy

1, Minimal: Provides no clear plan to confirm or exclude the differentials.

2, Competent: Outlines a reasonable plan to confirm the leading diagnosis and exclude the key alternatives.

3, Proficient: Specifies an efficient and risk-stratified diagnostic pathway with explicit decision points.

3.2 Therapeutic Planning

1, Minimal: Omits initial management or proposes something inappropriate or unsafe.

2, Competent: Proposes appropriate initial management for the leading diagnosis.

3, Proficient: Proposes guideline-concordant, prioritised management, including time-critical interventions and safe dosing.

3.3 Contingency Planning

1, Minimal: Provides no safety-netting, monitoring, or escalation guidance.

2, Competent: Provides basic safety netting and follow-up.

3, Proficient: Provides explicit monitoring, escalation triggers, and contingency steps for deterioration or treatment failure.

Domain 4: Reflection and Metacognition

4.1 Uncertainty Recognition

1, Minimal: Presents conclusions with unwarranted certainty and does not acknowledge uncertainty.

2, Competent: Acknowledges uncertainty and identifies the key unknowns.

3, Proficient: Calibrates confidence to the evidence and states what further data would resolve the uncertainty and how.

4.2 Cognitive Bias Awareness

1, Minimal: No awareness of potential reasoning pitfalls.

2, Competent: Notes at least one relevant bias or pitfall, such as anchoring or premature closure.

3, Proficient: Names the biases relevant to the case and describes concrete debiasing steps applied to its own reasoning.

4.3 Self-Directed Learning

1, Minimal: No knowledge gaps or next steps identified.

2, Competent: Identifies relevant knowledge gaps or resources.

3, Proficient: Identifies specific knowledge gaps and appropriate evidence sources and explains how new information would alter the plan.

Scoring rule

Raters and judges match the observed behaviour in the response to the written anchor, rather than forming a general impression. Scoring is based on what is present in the response. Length is not a proxy for quality: a brief response that meets the behavioural criteria scores 3, and a long response that does not scores lower.

References

1. Peterson BD, Magee CD, Martindale JR, et al. REACT: Rapid Evaluation Assessment of Clinical Reasoning Tool. J Gen Intern Med. 2022;37(9):2224-2229. doi:10.1007/s11606-022-07513-5.

2. Smith PC, Kendall LM. Retranslation of expectations: an approach to the construction of unambiguous anchors for rating scales. J Appl Psychol. 1963;47(2):149-155.
