# Supplementary material for: Process-Oriented, Behaviorally Anchored Assessment of Clinical Reasoning in Large Language Models and the Effect of Extended Thinking: Protocol for a Prospective, Multigroup, Comparative Study
Source: JMIR Res Protoc. 2026 Sep 22;15:e103220. doi: 10.2196/103220 (PMC13643257; doi:10.2196/103220)
Supplement: Supplementary file 4 [file resprot_v15i1e103220_app4.pdf]

Multimedia Appendix 4. LLM-as-Judge prompt templates and JSON schemas

Scoring is issued as two separate judge calls, so that the primary outcome cannot be influenced by the secondary instrument. The first call sees the Phase A response alone. The second call sees the Phase A and Phase B responses together. Both calls use the API at temperature 0.0 with structured JSON output, and both embed the full REACT-AI rubric (Multimedia Appendix 1) and the vignette. No reference answer is supplied in either call, which avoids an upward pull on the scores.

Call 1: REACT-AI and Disinformation Generation Rate (Phase A response only)

Prompt template (abridged). “You are an expert clinical-reasoning examiner. Using only the REACT-AI rubric below, score the candidate response. For each of the 13 sub-domains, first state the behavioural evidence you observed, then assign an integer score of 1, 2, or 3 strictly by matching that evidence to the written anchors. Do not reward general impressions, verbosity, or surface fluency. Do not assume facts that are not present in the response. Then list any clinically incorrect or fabricated statements. Output valid JSON only, with no text outside the JSON.”

Required JSON schema.

```
{
 "subdomain_scores": {
  "1.1": {"evidence": "string", "score": 1|2|3},
  "1.2": {"evidence": "string", "score": 1|2|3},
  "...": "...",
  "4.3": {"evidence": "string", "score": 1|2|3}
 },
 "domain_averages": {"1": number, "2": number, "3": number, "4": number},
 "total": number,
 "modified_score": number,
 "dgr_flags": ["string"]
}
```

Call 2: AI Metacognition Assessment Rubric (Phase A and Phase B responses)

Prompt template (abridged). “You are shown a candidate’s initial response and its subsequent self-correction. Using only the AI-MAR rubric below, score each of the five dimensions from 1 to 3 by matching observed behaviour to the written anchors. Consider spontaneous metacognition in the initial response and prompted metacognition in the self-correction. Do not re-score the clinical reasoning itself. Output valid JSON only, with no text outside the JSON.”

Required JSON schema.

```
{
 "ai_mar": {
  "uncertainty": {"evidence": "string", "score": 1|2|3},
  "error_detection": {"evidence": "string", "score": 1|2|3},
  "calibration": {"evidence": "string", "score": 1|2|3},
  "knowledge_boundary": {"evidence": "string", "score": 1|2|3},
  "process_awareness": {"evidence": "string", "score": 1|2|3}
 }
}
```


Panel rules

Two primary judges score each output under the conflict-of-interest rotation: no model family judges its own outputs, and judges are drawn from a different model tier or generation than the candidate. A tiebreaker is called only when the two primary judges differ by more than one point on any sub-domain, and the final sub-domain score is then the median of the three. The same disagreement rule and the same median resolution govern the human examiners, where a third examiner adjudicates while blind to the two primary scores. Inter-judge and inter-rater reliability are computed on the two primary scores, recorded before any tiebreaker or adjudication. A parallel shadow track, in which each model scores its own outputs, is retained separately to estimate the Self-Preference Bias Delta and is the single deliberate exception to blinding. The use of a panel of judges rather than a single evaluator, and the reporting of self-preference effects, follow current recommendations for LLM-as-Judge evaluation [1,2].

References

1. Zheng L, Chiang WL, Sheng Y, et al. Judging LLM-as-a-judge with MT-Bench and Chatbot Arena. Adv Neural Inf Process Syst. 2023;36:46595-46623.

2. Gu J, Jiang X, Shi Z, et al. A survey on LLM-as-a-judge. The Innovation. 2024;5(6).
