# Supplementary material for: Process-Oriented, Behaviorally Anchored Assessment of Clinical Reasoning in Large Language Models and the Effect of Extended Thinking: Protocol for a Prospective, Multigroup, Comparative Study
Source: JMIR Res Protoc. 2026 Sep 22;15:e103220. doi: 10.2196/103220 (PMC13643257; doi:10.2196/103220)
Supplement: Supplementary file 5 [file resprot_v15i1e103220_app5.pdf]

Multimedia Appendix 5. AI Metacognition Assessment Rubric (AI-MAR)

Five dimensions, each scored 1 (Minimal), 2 (Competent), or 3 (Proficient). AI-MAR is scored on the Phase A (unprompted) and Phase B (prompted self-correction) responses as a pair, so it captures metacognition that is both spontaneous and prompted by the task. It is a secondary instrument and is scored in a separate judge call from REACT-AI (Multimedia Appendix 3).

D1 Spontaneous uncertainty expression

1, Minimal: States conclusions without any unprompted acknowledgement of uncertainty.

2, Competent: Notes uncertainty where it exists without being asked.

3, Proficient: Flags uncertainty and its sources specifically and unprompted.

D2 Error detection

1, Minimal: Does not catch its own errors, even when prompted to do so.

2, Competent: Catches some of its own errors when prompted to self-correct.

3, Proficient: Catches and corrects its own errors, including subtle ones, and explains the corrections.

D3 Confidence calibration

1, Minimal: Confidence is poorly matched to correctness, whether overconfident or underconfident.

2, Competent: Confidence broadly tracks the strength of the evidence.

3, Proficient: Confidence is well calibrated and stated in actionable terms, for example, what would raise or lower it.

D4 Knowledge-boundary recognition

1, Minimal: Does not recognise the limits of its knowledge and may fabricate.

2, Competent: Recognises some limits and avoids over-claiming.

3, Proficient: Clearly marks what it does and does not know, and defers where appropriate.

D5 Reasoning-process awareness

1, Minimal: Shows no awareness of how it reached its conclusion.

2, Competent: Describes its reasoning steps at a general level.

3, Proficient: Spells out its reasoning, including the alternatives it considered and why it set them aside.

Relationship to REACT-AI

AI-MAR is reported alongside, and not merged into, the REACT-AI total. Domain 4 of REACT-AI (Reflection and Metacognition) scores metacognitive behaviour within the clinical reasoning task itself, whereas AI-MAR scores metacognitive behaviour across the unprompted and prompted turns as a pair. The association between the two is a pre-specified exploratory analysis. The metacognitive dimensions extend the Reflecting domain of the original REACT instrument [1] to text-based model outputs, and the anchors follow the same behaviourally anchored construction logic [2].

References

1. Peterson BD, Magee CD, Martindale JR, et al. REACT: Rapid Evaluation Assessment of Clinical Reasoning Tool. J Gen Intern Med. 2022;37(9):2224-2229. doi:10.1007/s11606-022-07513-5.

2. Smith PC, Kendall LM. Retranslation of expectations: an approach to the construction of unambiguous anchors for rating scales. J Appl Psychol. 1963;47(2):149-155.
